# Immune capacity determines outcome following surgery or trauma: a systematic review and meta-analysis

**DOI:** 10.1007/s00068-019-01271-6

**Published:** 2019-11-28

**Authors:** Ruiyi Jia, Moran Zhou, Camilla S. L. Tuttle, Andrea B. Maier

**Affiliations:** 1 Department of Medicine and Aged Care, @AgeMelbourne, Royal Melbourne Hospital, University of Melbourne, Melbourne, Australia; 2 Department of Human Movement Sciences, @AgeAmsterdam, Amsterdam Movement Sciences, Vrjie Universiteit, Amsterdam, Netherlands

**Keywords:** Complications, Innate immunity, Lipopolysaccharide, Surgery, Wounds and injuries

## Abstract

**Purpose:**

Immunological functions are altered following physical injury. The magnitude of the immunological response is dependent on the initial injury. However, variability in the immune response exists within and between patients where only some patients are at risk of developing complications such as systemic inflammatory response syndrome after injury. This systematic review and meta-analysis assessed whether lipopolysaccharide (LPS) induced cytokine production capacity of leucocytes can be used as a functional test to predict the risk of developing complications after injury.

**Methods:**

Medline, Embase and Web of Science were systematically searched to identify articles that investigated the association between LPS induced cytokine production capacity in leucocytes and any clinical outcome after surgery or trauma. Where sufficient information was supplied, a meta-analysis was performed to determine the overall clinical outcomes.

**Results:**

A total of 25 articles out of 6765 abstracts identified through the literature search were included in this review. Most articles described a positive association between cytokine production capacity and the development of inflammatory complications (*n* = 15/25). Coincidingly, the meta-analysis demonstrated that TNFα (Hedges *g*: 0.63, 95% CI 0.23, 1.03), IL-6 (Hedges *g*: 0.76, 95% CI 0.41, 1.11) and IL-8 (Hedges *g*: 0.93, 95% CI 0.46, 1.39) production capacity was significantly higher, one day after injury, in patients who developed inflammatory complications compared to patients who did not following trauma or surgical intervention. No significant difference was observed for IL-1β.

**Conclusion:**

The associations of elevated LPS-induced cytokine production capacity with the risk of developing inflammatory complications are consistent with previous theories that proposed excessive inflammation is accompanied by anti-inflammatory mechanisms that results in a period of immunosuppression and increased risk of secondary complications. However, immunological biomarkers for risk stratification is still a developing field of research where further investigations and validations are required.

**Electronic supplementary material:**

The online version of this article (10.1007/s00068-019-01271-6) contains supplementary material, which is available to authorized users.

## Background

Physical injury due to surgery or trauma induces a systemic inflammatory response [1, 2]. It is postulated that an inflammatory and anti-inflammatory response are simultaneously elicited following injury where the magnitude of the surgical stress is positively associated with the degree of inflammatory and anti-inflammatory response [1–4]. At the severe end of the inflammatory spectrum, the immunological responses can manifest as systemic inflammatory response syndrome (SIRS) or compensatory anti-inflammatory response syndrome (CARS), which are both associated with increased mortality and morbidity [1, 2, 5].

Multiple randomised controlled studies have investigated whether glucocorticoids given to trauma or surgical patients are effective in attenuating the post-operative or post-trauma immune response and subsequently decrease the risk of mortality and morbidity [6–8]. Despite evidence of effect in reducing molecular inflammatory parameters [9, 10], several meta-analysis’s have shown that glucocorticoids have no significant effect on decreasing adverse events after injury [7, 9, 11]. In fact, glucocorticoids were found to be associated with increased risk of gastrointestinal bleeding [7] and myocardial injury [8]. It has been proposed that the variation in immune response between patients may be partly responsible for the inconsistent effects of immunomodulation therapy [12]. Currently there is no risk stratification tool that has been established for evaluation of the risk of developing immune-associated complications after injury [12, 13].

Alterations in cytokine production capacity in response to lipopolysaccharide (LPS) after injury have been previously identified as potential prognostic biomarkers, however, its utility as a risk stratification tool has not been determined [13]. Therefore, this systematic review was conducted to evaluate whether LPS induced cytokine production capacity of blood-derived leucocytes from surgery or trauma patients can be used to predict risk of developing complications after injury. We hypothesized that cytokine production capacity can be used to risk stratify patients based on the assumption that there is variability in the immunological response induced after injury.

## Methods

### Data sources and search strategies

The protocol of the systematic review was registered at PROSPERO International prospective register of systematic reviews (PROSPERO registration no. CRD42018111288). This review adhered to the standards of the Preferred Reporting Items for Systematic Reviews and Meta-analysis (PRISMA). A systematic search across the databases Medline, Embase and Web of Science was performed from inception to the 4th of December 2017. Key search terms were designed using a combination of medical subject headings and keywords. The search was designed around terms such as “surgical procedure”, “wounds and injuries”, “cytokines” and “lipopolysaccharide” (refer to Supplementary Information 1 for complete search strategy). After removal of duplicates, the title and abstracts of returned articles were screened by two reviewers (R.J. and M.Z.) using the following inclusion and exclusion criteria. Articles were considered eligible, if (1) cytokine production capacity following ex vivo LPS stimulation of leucocytes in blood samples obtained from trauma or surgical patients at any time point was measured and associated with a clinical outcome, irrespective of the method of LPS stimulation utilized. (2) LPS stimulation was associated with a surrogate outcome measure such as the acute physiology and chronic health evaluation II score (APACHE II) [14] or multiple organ dysfunction score (MODS) [15]; and (3) a control group was included in the study design. Articles were excluded from this study if the articles included (1) participants who were pregnant (2) participants who had allogenic transplants, (3) participants who had had a systemic autoimmune disease, (4) participants who were receiving a form of immunosuppressive therapy, (5) participants who were diagnosed with an infection and (6) participants who were diagnosed with an outcome of interest e.g. SIRS or multiple organ dysfunction syndrome prior to study recruitment. After title and abstract screening, the full-text of the remaining articles was then screened for inclusion by two reviewers (R.J., M.Z.) using the above criteria. Disagreements between the two reviewers were arbitrated by a third reviewer (C.T.).

### Risk of bias

Articles were assessed for their risk of bias using the Newcastle–Ottawa Scale (NOS) following the guidelines and recommendation of the Cochrane Collaboration [16]. To accommodate this review, the NOS was adapted slightly for surgical populations (Supplementary Information II). Furthermore, because this review does not focus on the follow-up of studied populations Question 3 of the Outcome Assessment was not applicable to this review, resulting in an adjusted NOS score (Max—8 stars). The risk of bias for each study was assessed by two reviewers (R.J. and M.Z.) and any disagreements between the reviewers were arbitrated by a third reviewer (C.T.).

### Data collection and analysis

Data describing participant characteristics, the methodology of ex vivo LPS stimulation assays, cytokine production and clinical outcome measures were extracted by two reviewers (R.J. and M.Z.) using a standardized data extraction form. Cytokine concentrations were extracted from figures if no numerical values were presented. Cytokine production capacity was defined as concentration of cytokine produced after LPS stimulation minus the concentration of cytokine produced without LPS stimulation. Inflammatory complications were defined as a composite of SIRS/sepsis or other infectious complications. Where available, the injury severity score (ISS) was extracted for trauma populations [17]. Data was pooled if a study only provided sub grouped analysis of the outcome and uneventful groups. The pooled standard deviation was derived using the following formula.

$${\text{spooled}} = \sqrt {\frac{{\left( {n_{1} - 1} \right)s_{1}^{2} + \left( {n_{2} - 1} \right)s_{2}^{2} }}{{n_{1} + n_{2} - 2}}} .$$

### Statistical methods

To determine the overall ability of LPS to predict surgical and trauma outcomes a meta-analysis, using the comprehensive meta-analysis (CMA) Software (Biostat, Englewood, New Jersey, USA) was performed. As such the following data were also extracted; sample size, mean difference in cytokine production capacity after LPS stimulation in patients who developed complications compared to patients who did not, standard deviation, *p* value and statistical test. Due to the large variability in the LPS stimulation technique utilised by the articles to induce cytokine production, standardized difference in means (SMD) was used to represent the difference in cytokine production capacity. Specifically, Hedges’ *g* was used to compute the SMD to obtain a more conservative estimate of the effect size as most studies included a small sample size. A random effects model was used for all pooled data analyses. Study heterogeneity was assessed using Cochran’s *Q* and *I*^2^ test, where *p* < 0.1 was considered significant [18]. Publication bias was evaluated if more than two articles investigated the same outcome. Due to the limited number of articles incorporated in the meta-analysis, the funnel plot was only inspected graphically for an approximation [19].

## Results

**Fig. 1:**
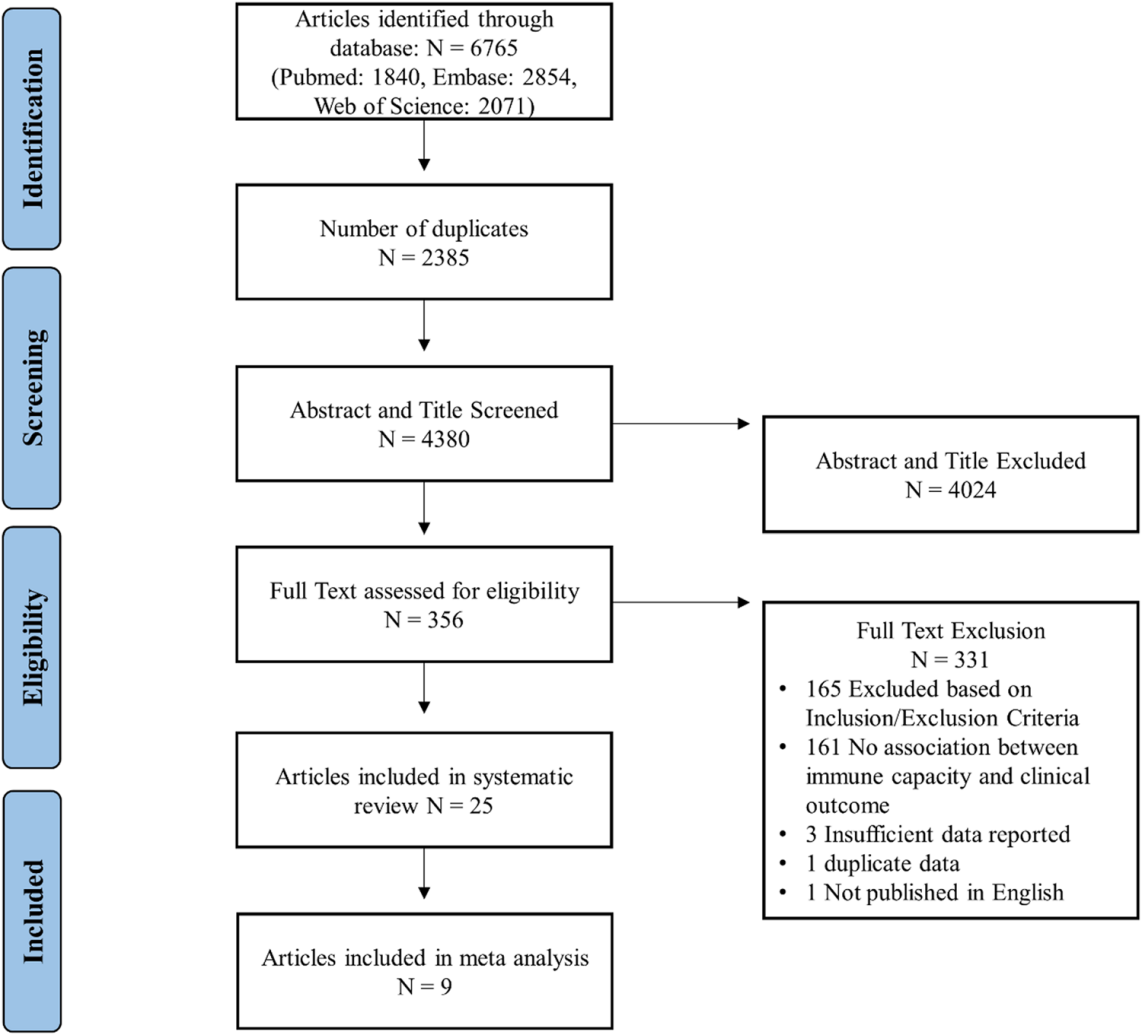
Overview of the search strategy

In total, 6765 articles were isolated in the initial search, after removal of duplicates 4380 articles were screened by title and abstract of which 356 articles were screened by full text. Figure 1 describes the overall search strategy. Overall, 25 articles were included in this review of which 9 were eligible for inclusion in the meta-analysis.

Table 1 describes the characteristics of a total of 1350 patients analysed in the 12 surgical (6 cardiac, 4 cancer, 2 abdominal) and 13 trauma articles reviewed. The majority of articles (*n* = 10/13) in trauma populations reported an injury severity score (ISS) and all articles utilised populations suffering from major trauma with an average ISS > 15 points. The majority of studies collected whole blood samples in heparin vacutainers and used Roswell Park Memorial Institute medium 1640 (RPMI-1640) as the blood diluting agent. The most commonly used LPS was derived from *Escherichia coli *(*E. coli*)*.* However, the studies varied widely in overall methodology utilising a variety of different bacterial strain, LPS concentrations (range 0.01–1000 ng/ml) and duration of blood sample incubation with LPS (2–24 h) to stimulate an immune response. For further information on experimental procedures refer to Supplementary Table 1.

**Table 1:** Characteristics of patients undergoing surgery or after a trauma

First author, year	Type of surgery/ISS	Total and subgroups	*N*	Age (year)	Female (%)
Surgery patients studies					
Justus, 2017 [48]	Cardiac surgery	T	20	0.41 (0.19–3.18)	40
		O: Ventilation time	20	0.41 (0.19–3.18)	40
		C: N/A			
Flier, 2015 [20]	Cardiac surgery	T	84	66.8 ± 1.0^a^	21.4
		O: Inflamm. (SIRS or pneumonia)	19	63.7 ± 2.2^a^	15.8
		C: UE	65	67.7 ± 1.1^a^	23.1
Stoppelkamp, 2015 [49]	Cardiac surgery	T	10	65.6 ± 4.3^b^	20
		O: SIRS	5	61.4 ± 7.1^a^	0
		C: UE	5	69.8 ± 4.7^a^	40
Kumpf, 2010 [22]	Cardiac surgery	T	415	66.5 ± 0.6^a^	26.5
		O: SIRS or sepsis	NR	NR	NR
		C: UE	NR	NR	NR
Allen, 2006 [50]	Cardiac surgery	T	36	0.54 (0.0–2.0)	41.7
		O 1: ICU LOS > 5 d	11	NR	NR
		C 1: ICU LOS ≤ 5 d	25	NR	NR
		O 2: Sepsis/mortality	4	NR	NR
		C 2: UE	32	NR	NR
Tashiro, 2001 [51]	Cardiac surgery	T	34	66.1 ± 1.6^b^	29.4
		O: Restenosis	14	66.9 ± 2.1	28.6
		C: UE	20	65.5 ± 2.2	30
Jones, 2014 [27]	Cancer thoracic surgery	T	40	66.9 ± 1.2^a^	42.5
		O: Pneumonia	14	NR	NR
		C: UE	26	NR	NR
Van Bokhorst, 2000 [52]	Cancer head and neck surgery	T	49	58.3 ± 1.5^b^	38.8
		O: Mortality	32	58 ± 1.8^a^	46.9
		C: UE	17	59 ± 2.7^a^	23.5
Mokart, 2010 [53]	Cancer gastrointestinal surgery	T	19	56.7 ± 2.5^b^	NR
		O: Sepsis	7	58.0 ± 2.9^a^	NR
		C: UE	12	56.0 ± 4.5^a^	NR
Spies, 2004 [54]	Cancer gastrointestinal surgery	T	54	NR	13
		O: Infectious complication	23	NR	NR
		C: UE	31	NR	NR
Riese, 2000 [21]	Abdominal surgery	T	50	60.5 ± 1.8^b^	32
		O: Inflamm. (Pneumonia, intra-abdominal abscess, SIRS)	9	NR	NR
		C: UE	41	NR	NR
Ziegenfuss, 1999 [55]	Abdominal surgery	T	14	68.2 ± 2.5	NR
		O: APACHE II	14	68.2 ± 2.5	NR
		C: N/A			
Trauma patients studies					
Paraschos, 2015 [26]	23.15 ± 1.2^a^	T	69	41 ± 2.3^a^	15.9
		O: Sepsis mortality	8	NR	NR
		C: Sepsis survivor	28	NR	NR
Relja, 2015 [28]	29.9 ± 1.8	T	30	40.5 ± 3.1	33.3
		O: Sepsis	6	NR	NR
		C: UE	24	NR	NR
Kirchhoff, 2009 [56]	32 ± 3.0^c^	T	13	41 ± 5.0	30.8
		O: MODS score	13	41 ± 5.0	30.8
		C: N/A			
Wutzler, 2009 [30]	25.7 ± 2.3^b^	T	58	43 ± 3.0	31
		O 1: SIRS or sepsis	35	40.9 ± 2.9	42.9
		C 1: UE	23	49.7 ± 5.1	13
		O 2: Mortality	7	57.7 ± 10.3	42.9
		C 2: UE	7	53.4 ± 6.9	28.6
Ploder, 2006 [25]	40.6 ± 2.5^a^	T	19	38.6 ± 3.4^a^	15.8^d^
		O: Sepsis mortality	6	50.3 ± 8.9^a^	16.7^d^
		C: Sepsis survivor	13	33.2 ± 3.0^a^	7.7^d^
Laudanski, 2004 [57]	NR	T	76	42.2 ± 2.8^a^	38.2
		O: MODS score	76	42.2 ± 2.8^a^	38.2
		C: N/A			
Spolarics, 2003 [24]	25.8 ± 0.9	T	12	34.1 ± 3.8	0
		O 1: ARDS	3	NR	0
		C 1: UE	NR	NR	0
		O 2: Sepsis	5	NR	0
		C 2: UE	NR	NR	0
		O 3: BFI	3	NR	0
		C 3: UE	NR	NR	0
Heesen, 2002 [58]	27 ± 2.3^b^	T	57	38 ± 4.6^b^	36.8
		O: Sepsis	14	NR	NR
		C: UE	43	NR	NR
Majetschak, 2000 [45]	27 ± 1.1^a^	T	84	38 ± 1.6^a^	29.8
		O: Sepsis	23	46 ± 3.5^b^	30.4
		C: UE	61	35 ± 1.8^b^	29.5
Majetschak, 2000 [23]	33 ± 1.9	T	32	38 ± 3.0^a^	31.3
		O: Sepsis/MOF	10	NR	NR
		C: UE	22	NR	NR
Flach, 1999 [59]	26.0 ± 1.2^b^	T	40	36.0 ± 1.8^b^	42.5
		O: Sepsis	10	43 ± 3.5^a^	30
		C: UE	30	33.7 ± 2.2^a^	46.7
Schluter, 1991 [60]	NR	T	12	45.5 ± 5.0^b^	8.3
		O: Sepsis mortality	7	43 ± 19	0
		C: Sepsis survivor	5	45 ± 12	20
Wood, 1984 [29]	NR	T	23	48.9 (22–91)	30.4
		O: Sepsis	9	43.3 ± 7.4^a^	0
		C: UE	14	51.7 ± 4.8^a^	50

Values represent mean ± SEM or median (range) unless otherwise specified

*N* sample size, *UE* uneventful which refers to patients who did not develop the outcome of interest, *N/A* not applicable, *NR* not reported, *ICU LOS* intensive care unit length of stay, *ISS* injury severity score, *T* total, *O* outcome, *C* comparator. *Inflamm.* inflammatory complication, *d* days, *SIRS* Systemic inflammatory response syndrome, *APACHE II* Acute physiology and chronic health evaluation II score, *MODS* Multiple organ dysfunction syndrome, *ARDS* Adult respiratory distress syndrome, *BFI* body fluid infection, *MOF* multiple organ failure

^a^SEM recalculated using sample standard deviation

^b^Values recalculated by pooling subgroup values

^c^Scored using the new injury severity score

^d^Error in values reported as total females in subgroups did not match the reported total number of females

**Fig. 2:**
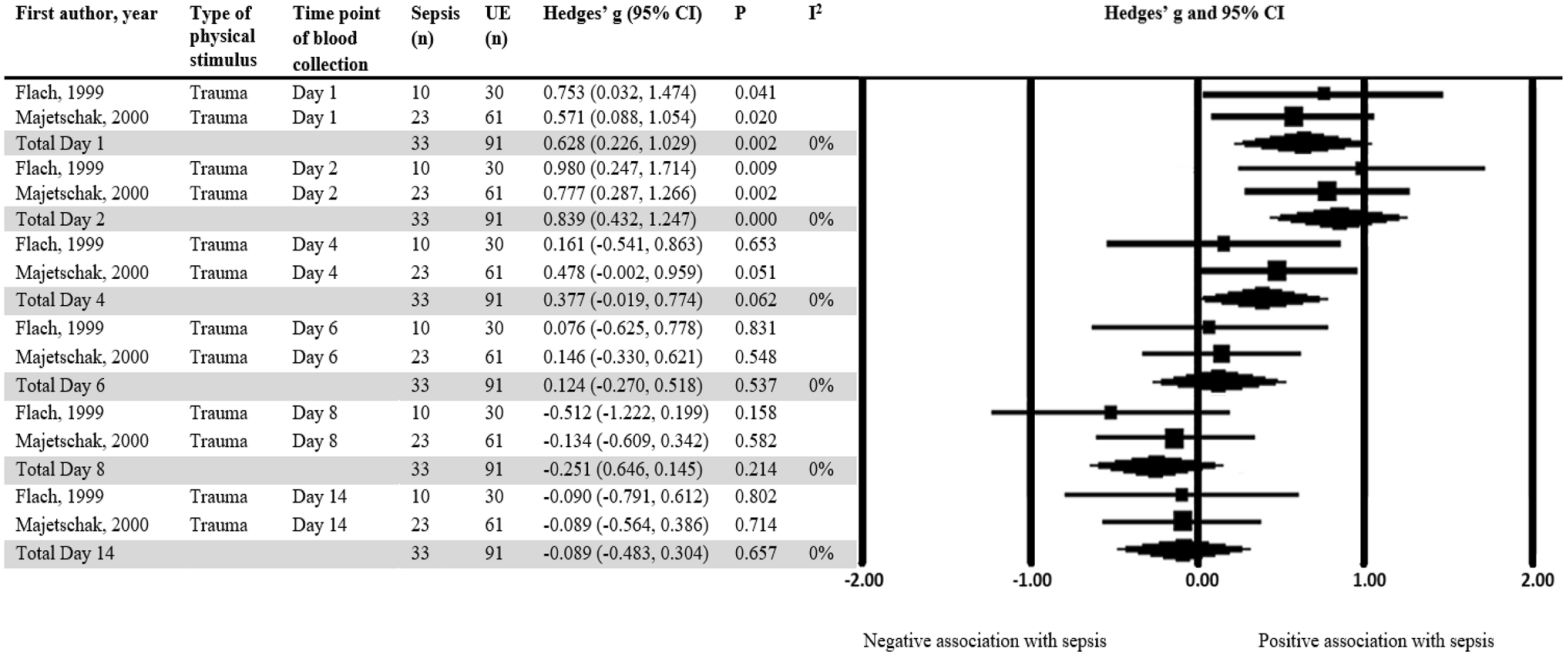
Standardized difference in means of TNFα production capacity after LPS stimulation in patients with and without sepsis development. A mean difference of < 0 indicates a lower cytokine production capacity in the septic group compared to the uneventful group. Day 1 to day 14 are in reference to the time of admission to hospital. *N* refers to the total number of patients. *UE* uneventful, *CI* confidence interval, *Df* degrees of freedom

**Fig. 3:**
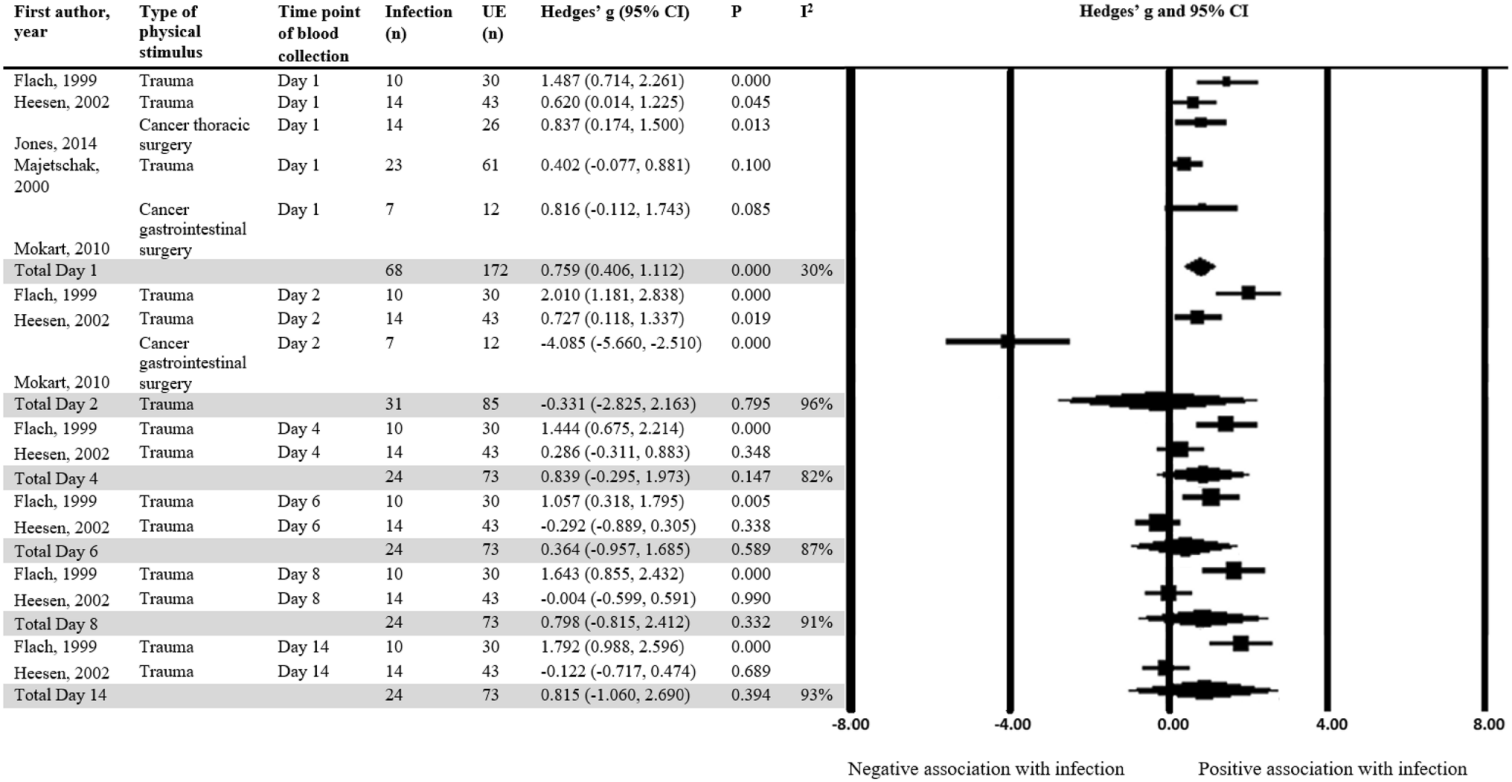
Standardized difference in means of IL-6 production capacity after LPS stimulation in patients with and without infectious complications. A mean difference of < 0 indicates a lower cytokine production capacity in the infection group compared to the uneventful group. Day 1 to day 14 are in reference to the time of hospital admission for trauma patients or post-operative days for surgery patients. In this case, infection is defined as sepsis or other infectious complications. N refers to the total number of patients. *UE* uneventful, *CI* confidence interval, *Df* degrees of freedom

**Fig. 4:**
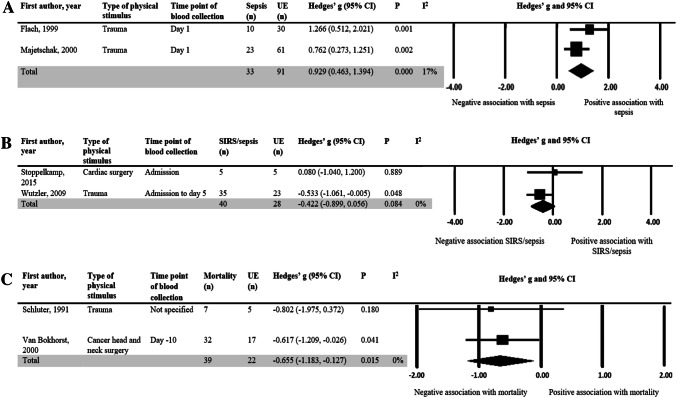
Standardized difference in means of IL-8, IL-1β and IL-6 production capacity after LPS stimulation. *N* refers to the total number of patients. *UE* uneventful, *CI* confidence interval, *Df* degrees of freedom. **a** A mean difference of < 0 indicates a lower cytokine production capacity in the septic group compared to the uneventful group. Day 1 refers to one day after hospital admission. **b** A mean difference of < 0 indicates a lower cytokine production capacity in the SIRS/sepsis group compared to the uneventful group. Admission refers to on admission into hospital. Values for Wutzler, 2009 was expressed as mean low IL-1β concentration from admission day to day 5. **c** A mean difference of < 0 indicates a lower cytokine production capacity in the mortality group compared to the uneventful group. Day − 10 refers to 10 days prior to the day of surgery

Table 2 describes the associations between cytokine production and patient outcome. Overall, 9 different clinical outcomes have been reported in the 25 articles. The two most commonly investigated clinical outcomes were inflammatory complications (*n* = 17) and mortality (*n* = 4). The association of cytokine production capacity with other clinical outcomes included MODS score (*n* = 2), ventilation time, composite outcome of sepsis or mortality, adult respiratory distress syndrome (ARDS), APACHE II score, restenosis and length of stay (LOS) in the intensive care unit. By grouping the studies according to the clinical outcome and type of cytokine measured, meta-analysis was performed on studies that investigated the association of TNFα (Fig. 2), IL-6 (Fig. 3), IL-8 (Fig. 4a) and IL-1β (Fig. 4b) with inflammatory complications. While Fig. 4c shows the associations between IL-6 and mortality.

**Table 2:** Association between cytokine production and outcome variables

First author, year	Type of surgery	Cytokines studied	Time point of blood collection	Outcome	Comparator	Main findings
Justus, 2017 [48]	Cardiac surg	TNFα	Pre, post, 4 h	Ventilation time	N/A	ATP: − −
Flier, 2015 [20]	Cardiac surg	TNFα, IL-6, IL-8	Pre, end of CPB, post, d1	Inflamm	UE	∆AC (post–pre): NS; OR per 10% increase in each cytokine concentration: NS; Adjusted (age, gender, anaesthesia, type) OR: NS
Stoppelkamp, 2015 [49]	Cardiac surg	IL-1β	Ad, pre, post, d1-3, d5, d8	SIRS	UE	d1: ± ; OTP: NS
Kumpf, 2010 [22]	Cardiac surg	TNFα, IL-6, IL-10	Pre, 4–6 h, d1-3	SIRS or sepsis	UE	AC: NS (QDNS)
Allen, 2006 [50]	Cardiac surg	TNFα, IL-6, IL-10	Pre, aortic cross-clamp release, end of CPB, after modified ultrafiltration, ICU Ad	ICU LOS > 5 d	ICU LOS ≤ 5d	IL-6 and IL-10 (post): −; TNFα (post): ± ; TNFα or IL-10 > 100 pg/ml (post): NS
		TNFα, IL-10	Ad, 2 h, 8 h, 24 h	Sepsis/mortality	UE	TNFα and IL-10 (ATP): − −
Tashiro, 2001 [51]	Cardiac surg	TNFα, IL-1α, IL-1β, IL-6, IFNγ, G-CSF	Pre, 3–6 m follow up	Restenosis	UE	AC (ATP): NS
Jones, 2014 [27]	Cancer thoracic surg	TNFα, IL-1β, IL-6, IL-8, IL-10, IL-12	Pre, 6 h, d1, d2	Pneumonia	UE	IL-6 (d1, d2): + ; IL-8 (d2): + ; IL-10 (d1): + ; IL-12 (pre): − −; IL-6, IL-8, IL-10 and IL-12 (OTP), and TNFα and IL-1β (ATP): NS; Pneumonia RR (cut-off: 414 pg/ml/µg IL-6, 1.988 pg/ml/µg IL-10, 0.147 pg/ml/µg IL-12): NS
Van Bokhorst, 2000 [52]	Cancer head and neck surg	TNFα, IL-6	Pre, d1, d4, d7, d10	Mortality	UE	Both cytokines (d10): −
Mokart, 2010 [53]	Cancer GI surg	IL-1Ra, IL-10, IL-6, IL-12p40	Pre, d1, d2, d3, d7	Sepsis	UE	IL-12p40: − (d1-d3), NS (pre, d7); OC (ATP): NS
Spies, 2004 [54]	Cancer GI surg	IFNγ/IL-10	Pre, d1, d3, d5, d7	Infectious complication	UE	IFNγ/IL-10 ratio (d1): −
Riese, 2000 [21]	Abd. surg	TNFα, IL-6	Pre, 6 h, d3	Inflamm	UE	Both cytokines (ATP): NS
Ziegenfuss, 1999 [55]	Abd. surg	TNFα, IL-1, IL-6, IL-10	Pre, EOI, 1.5 h after declamping, 24 h	Mean APACHE II from d1-d4	N/A	TNFα (EOI): −; TNFα (OTP) and OC (ATP): NS
Paraschos, 2015 [26]	Trauma	TNFα, IL-10, IL-17, IFNγ	D1 (Ad), d1 (Sepsis)	Sepsis mortality	Sepsis survivor	ΔTNFα (Ad d1—Sepsis d1): + + ; OC (ATP): NS
Relja, 2015 [28]	Trauma	IL-1β	Ad, d1-d10	Sepsis	UE	IL-1β at ATP: NS (QDNS)
Kirchhoff, 2009 [56]	Trauma	TNFα, IL-1β, IL-6, IL-8	Ad, 6 h, 12 h, d1, d2, d3	MODS score	N/A	% of CD14 + cells ex TNFα, IL-1β, IL-6 and IL-8: − − −
Wutzler, 2009 [30]	Trauma	IL-1β	Ad, d1-d5	SIRS or sepsis	UE	Mean low IL-1β (Ad-d5): −
				Mortality	UE	Mean low IL-1β (Ad-d5): ±
Ploder, 2006 [25]	Trauma	TNFα	D1-14	Sepsis mortality	Sepsis survivor	- (d1, d3, d5, d6, d10, d11, d13), − − (d2, d4), NS (OTP)
Laudanski, 2004 [57]	Trauma	mTNFα	Within 3d of Ad, 2/wk until ICU discharge	MODS score	N/A	mTNFα: + +
Spolarics, 2003 [24]	Trauma	TNFα, IL-10, IL-12	D2, d5, d10	Adult respiratory distress syndrome	UE	% mono ex IL-10 and IL-12 (d2): −; % mono ex TNFα (d2): NS
				Sepsis/SIRS	UE	% mono ex TNFα and IL-12 (d2) corr with sepsis: −; % mono ex IL-12 (d2) corr with sepsis and SIRS duration: −; % mono ex IL-10 (d2) corr with sepsis: NS
				Body fluid infection	UE	IL-6: + (d1, d2), NS (OTP)
Heesen, 2002 [58]	Trauma	IL-6	D1, d2, d4, d6, d8, d14	Sepsis	UE	+ (d1, d2); NS (OTP)
Majetschak, 2000 [45]	Trauma	TNFα, IL-6, IL-8	D1, d2, d4, d6, d8, d14	Sepsis	UE	TNFα: + (d1), NS (OTP); IL-6 (d1): + ; IL-8 (d1): + +
Majetschak, 2000 [23]	Trauma	TNFα	Ad, d1, d2, d4, d6, d8, d14	Sepsis or multiple organ failure	UE	TNFα: NS (QDNS)
Flach, 1999 [59]	Trauma	TNFα, IL-6, IL-8	Ad, d1, d2, d4, d6, d8, d14	Sepsis	UE	TNFα (Ad, d1), IL-6 and IL-8 (Ad, d1, d2): + ; TNFα, IL-6 and IL-8 (OTP): NS
Schluter, 1991 [60]	Trauma	IL-6	2/wk for 50 d/until ICU discharge	Sepsis mortality	Sepsis survivor	IL-6: NS (time point not specified)
Wood, 1984 [29]	Trauma	IL-1	Ad, 2/wk until discharge/death	Sepsis	UE	IL-1: NS (QDNS)

Where more than one outcome was investigated, the outcomes are listed sequentially with their corresponding comparator group. Day (d) and hour (h) numbers in the column, time point of blood collection, are in reference to time after surgery for surgical patients or time of admission to hospital for trauma patients. All references to cytokines in the main findings column refer to cytokine concentrations after LPS stimulation. Time points that were not mentioned in the main findings column but were investigated in the study are time points where data was not available

*Surg* surgery, *N/A* not applicable, *Pre* pre-operative period, *Post* post-operative period, *ATP* all time points, *Inflamm.* inflammatory complication, *UE* uneventful which refers to patients who did not develop the outcome of interest, *CPB* cardiopulmonary bypass, *AC* all cytokines, *NS* non-significant, *OR* odds ratios, *SIRS* systemic inflammatory response syndrome, *OTP* other time points, *QDNS* quantitative data not shown, *ICU* intensive care unit, *LOS* length of stay, *m* months, *RR* relative risk, *mTNFα* membrane-associated TNFα, *GI* gastrointestinal, *Abd.* Abdominal, *Ad* admission, *EOI* end of ischemia, *2/wk* twice per week, *OC* other cytokines, *corr* correlation, *ex* expressing, *mono* monocytes. ±, 0.05 < *p* < 0.01; +, *p* < 0.05; +  +, *p* < 0.01; +  +  +, *p* < 0.001; −, *p* < 0.05; − −, *p* < 0.01; − − −, *p* < 0.001. Sign indicates direction of cytokine change from comparator group to outcome group

A significantly higher TNFα production capacity was observed on day one (Hedges’ *g* = 0.63, 95% CI 0.23, 1.03) and two (Hedges’ *g* = 0.84, 95% CI 0.43, 1.25) after injury in patients who developed inflammatory complications compared to the uneventful group (Fig. 2). No statistical significance was present between the groups on day 4, 6, 8 and 14. Five studies [20–24] could not be incorporated into the meta-analysis (Table 2). Of these, four studies [20–23] found no significant differences in TNFα production capacity and inflammatory complications (e.g. SIRS, sepsis and pneumonia), whereas one study [24] reported a significantly lower percentage of monocytes expressing TNFα in patients who developed inflammatory complications compared to patients who did not.

Two studies that assessed whether the production capacity of TNFα was associated with mortality could not be included in a meta-analysis. One study [25] reported a significantly lower TNFα production capacity in patients who died following sepsis while another [26] reported a significantly higher change in TNFα production capacity on the first day of sepsis from baseline in septic patients who died compared to patients who survived (Table 2).

Figure 3 shows the mean IL-6 production capacity at day one after injury was significantly higher in patients who developed inflammatory complications (Hedges’ *g* = 0.76, 95% CI 0.41, 1.11). This difference in IL-6 production capacity was non-significant on day 2 to day 14. No significant associations between IL-6 production capacity and inflammatory outcomes were reported by three other studies [20–22] where insufficient data was provided for inclusion in the meta-analysis (Table 2).

The mean IL-8 production capacity was significantly higher one day after injury in patients who developed inflammatory complications compared to patients who did not as shown in Fig. 4a (Hedges’ *g* = 0.93, 95% CI 0.46, 1.39). Two other studies [20, 27] also investigated the relationship between IL-8 production capacity and development of inflammatory complications. One study [20] observed a non-significant odds ratio per 10% increase in relative change of IL-8 post-operatively from pre-operatively. Another study [27] found a significantly higher IL-8 production capacity in patients who developed pneumonia compared to patients who did not (Table 2).

No significant difference in means was observed for IL-1β production capacity in patients who developed an inflammatory complication compared to patients who did not (Fig. 4b). This is consistent with the findings of two other articles [28, 29] who reported no association of IL-1 production capacity with inflammatory outcomes (Table 2**)**. Of particular note is that the only study [30] that reported a significant negative relationship between IL-1β and inflammatory outcomes reported their cytokine concentrations as the average low IL-1β concentration from admission to five days after.

Figure 4c demonstrates the standardized difference in means of IL-6 production capacity in patients who died compared to patients who did not. The meta-analysis shows that IL-6 production was 0.66 (95% CI 0.13, 1.18) standard deviations lower in patients who subsequently died after surgery or trauma.

A summary table of the study quality using a modified NOS is presented in Supplementary Table 2. Overall, the adjusted mean number of stars assigned to the studies was 4.64 with a range of 2–7. This indicates that most included studies were of a reasonable quality. Visual inspection of the funnel plot of the 5 studies included in the meta-analysis for IL-6 appears symmetrical (Supplementary Fig. 1) and, as such, indicates low publication bias.

## Discussion

The development of inflammatory complications in patients after day one of injury (surgery/trauma) was significantly associated with a higher LPS induced production of TNFα, IL-6 and IL-8 compared to uneventful patients. These results are consistent with previous theories that proposed alterations in inflammatory and anti-inflammatory mechanisms can result in a period of immunosuppresion during which patients are at an increased risk of secondary infections [31]. However, the precise immunological alterations induced by physical injury and the complex immunoregulation that follows is still not well understood [2, 32]. Interestingly, no significant difference in IL-1β production capacity was observed between patients who developed inflammatory complications and patient who did not, suggesting that not all pro-inflammatory cytokines play a role in this response. The degree of the initial inflammatory response is dependent on the magnitude of tissue injury [33, 34]. Therefore, the elevation in pro-inflammatory cytokine production capacity observed in patients who later developed complications may indicate the severity of physical injury, rather than immunosuppression. Furthermore, it has also been proposed that cytokine production capacity after stimulation is at least partially determined by genetics, which means some individuals may be genetically predisposed to developing inflammatory complications [35]. For example, the application of this immunological LPS function test is not restricted to only surgery or trauma patients and in fact, it has been found to be predictive of mortality in community dwelling geriatric populations [36].

In this review, most studies only identified a difference in cytokine production capacities between the outcome and uneventful group during the first two days after trauma or surgery. Effectively, this suggests that there is a restricted timeframe in which cytokine production capacity can be utilised for prognostic testing in patients with physical injury. Other conventional immunological markers such as C-reactive protein have also been found to be predictive of infectious complications after surgery, however, they are often only predictive after post-operative day three to four [37, 38] by which time prophylactic treatment may be futile.

Given the dynamic nature of the immune system, there is substantial interperson and intraperson variability in LPS induced cytokine capacity [39]. Many factors other than the severity of the initial physical injury have been found to influence the cytokine response elicited after LPS stimulation. Cytokine production capacity has been found to differ with age [40], gender [41] and to a lesser extent anaesthetics [42], blood transfusion [43] and diet [44]. However, out of the articles reviewed, only Flier [20] and Majetschak [45] have accounted for age and gender in their interpretation of cytokine production capacities. Priming of leucocytes with bacterial components have also been associated with alterations in leucocyte production capacity of cytokines [46], however, its effect has been minimised, but not entirely accounted for, by excluding patients with chronic or active infection. Potentially, these variables may be confounding factors which masks the associations between cytokine production capacity and clinical outcomes reported by the studies analysed in this review.

Immunological biomarkers for risk stratification is a developing field of research. As shown in this review, there are a limited number of studies that have investigated the relationship between cytokine production capacity and clinical outcome. This could be due to the lack of a standardised methodology for LPS induced cytokine production capacity. Specifically, whether whole-blood or isolated monocytes is the most appropriate specimen to perform LPS induced cytokine capacity tests appears to be contentious. Given that *E. coli* derived LPS is likely to stimulate a TLR4 response of the monocytes it would appear that a standardised approach using a predetermined LPS concentration for a specific number of seeded monocytes is likely to reduce some inter-individual variation. Furthermore, most of the studies reviewed focused on exploring the association between cytokines such as TNFα, IL-6 and IL-8, while only a few studies have examined other pro-inflammatory cytokines such as IL-1, or anti-inflammatory cytokines such as IL-10 following injury. The inclusion of more cytokines would provide a more comprehensive profile of the inflammatory response following injury. Similarly, non-inflammatory complications after surgery or trauma have only been investigated by a few studies and are often based on small sample sizes. Therefore, due to the paucity of information currently available, it is not feasible to reach a conclusion as to whether cytokine production capacity is predictive of non-inflammatory complications.

To our knowledge, this is the first systematic review that has investigated whether LPS induced cytokine production capacity in leucocytes can differentiate patients who are at risk of developing an adverse event after injury. By incorporating both surgical and trauma patients, it provides a broad overview of the associations of cytokine production capacity with clinical outcome after injury. However; this review is not without limitations. Many studies that reported a non-significant association between cytokine production capacity and the subsequent clinical outcome that developed, did not publish enough data for inclusion in the meta-analysis. As such this is likely to have impacted the overall findings. For example, while 7 studies have investigated the predictive capability of TNFα production capacity for inflammatory complications only 2 studies were included in the meta-analysis. Most of the studies (4/5) not included in the meta-analysis found that there was no significant difference in TNFα production capacity between the measured outcomes. Although no plot asymmetry was apparent in the funnel plots generated, it is likely publication bias does exist in this review given it has been suggested that at least 10 studies are required to obtain a reasonable statistical analysis of plot asymmetry [19]. Another factor is that not all studies reported a definition for their outcome. Also most studies included in this review were based on major surgical or trauma patients, whether the findings of this review can be extrapolated to patients after minor injury or surgical interventions is unknown. Furthermore, aside from *E. coli* derived LPS the methodology employed by the studies to induce cytokine production ex vivo varied widely. The absence of a standardized ex vivo LPS stimulation method and reporting system impedes the determination of the absolute difference in cytokine concentration after LPS stimulation in the outcome compared to the uneventful group. Consequently, results had to be expressed as standardized difference in means (SMD). Therefore, depending on the cause of the variation in standard deviation between studies, the SMD may be an over or under estimation of the true effect [47].

## Conclusion

TNFα, IL-6 and IL-8 production capacities at day one after injury were higher in trauma or surgical patients who developed inflammatory complications compared to patients who did not. No significant differences were observed for IL-1β. Evidence for the association between cytokine production capacity and other clinical outcomes is currently limited and requires further investigation. Subsequently, standardisation and validation of the sensitivity and specificity of this prognostic test is required.

## Electronic supplementary material

Below is the link to the electronic supplementary material.

Supplementary file1 (DOCX 186 kb)

Supplementary file2 (TIFF 44 kb)
