# Supplementary material for: Immune capacity determines outcome following surgery or trauma: a systematic review and meta-analysis
Source: Eur J Trauma Emerg Surg. 2019 Nov 28;46(5):979–91. doi: 10.1007/s00068-019-01271-6 (PMC7593308; doi:10.1007/s00068-019-01271-6)
Supplement: Supplementary file 1 — Supplementary file1 (DOCX 186 kb) [file 68_2019_1271_MOESM1_ESM.docx]

## **Supplementary Information I:** Search Strategy

Medline (1946 to present) search method

1. Exp Surgical Procedures, Operative/
2. Exp “Wounds and Injuries”/
3. (lipopolysaccharide* or endotoxin*).mp
4. Su.fs
5. (surg* or cardiopulmonary bypass* or laparo* or trauma or injur* or fracture*).mp
6. Exp Cytokines/
7. (cytokine* or tumo?r necrosis factor* or interleukin* or chemokine* or interferon* or transforming growth factor*).mp
8. 1 or 2 or 4 or 5
9. 8 and 3 and (6 or 7)
10. (rat or rats or mouse or mice or rabbit or rabbits or pig or pigs).mp
11. Limit 9 to animals
12. Limit 9 to English language
13. Limit 12 to “review”
14. 12 not (10 or 11 or 13)

Embase (1947 to 1/12/2017) search method

1. Exp Surgical Procedures, Operative/
2. Exp “Wounds and Injuries”/
3. (lipopolysaccharide* or endotoxin*).mp
4. Su.fs
5. (surg* or cardiopulmonary bypass* or laparo* or trauma or injur* or fracture*).mp
6. Exp Cytokines/
7. (cytokine* or tumo?r necrosis factor* or interleukin* or chemokine* or interferon* or transforming growth factor*).mp
8. 1 or 2 or 4 or 5
9. 8 and 3 and (6 or 7)
10. (rat or rats or mouse or mice or rabbit or rabbits or pig or pigs).mp
11. Limit 9 to animals
12. Limit 9 to English language
13. Limit 12 to conference abstracts
14. Limit 12 to “review”
15. 12 not (10 or 11 or 13 or 14)

Web of Science core collection

1. TS=(surg* or cardiopulmonary bypass* or laparo* or trauma or injur* or fracture*)
2. TS=(lipopolysaccharide* or endotoxin*)
3. TS=(cytokine* or tumo?r necrosis factor* or interleukin* or chemokine* or interferon* or transforming growth factor*)
4. TS=(rat or rats or mouse or mice or rabbit or rabbits or pig or pigs or animal or animals)
5. #1 AND #2 AND #3
6. #5 not #4
7. #6 AND LANGUAGE: (English) Refined by: [excluding] DOCUMENT TYPES: (REVIEW OR MEETING ABSTRACTS OR BOOK CHAPTER)

**Supplementary Information II:** Adjusted Newcastle Ottawa Quality Assessment Scale – Cohort Studies **Selection**
1) Representativeness of the exposed cohort
 a) Inclusion of consecutive trauma or surgical patient admitted to the hospital
 b) Inclusion of consecutive trauma or surgical patient admitted to the hospital with
 additional inclusion and exclusion criteria applied
 c) selected group of users eg nurses, volunteers
 d) no description of the derivation of the cohort
2) Ascertainment that patients were excluded if they have taken immunosuppressants, suffers
 from systemic autoimmune disease, had an allogenic transplant or were infected at the start
 of the study
 a) Stated exclusion of all the criteria above
 b) Stated exclusion of some of the criteria above
 c) No description of exclusion criteria
3) Ascertainment of exposure
 a) secure record (eg surgical records)
 b) structured interview
 c) written self report
 d) no description

4) Demonstration that outcome of interest was not present at start of study
 a) yes
 b) no **Comparability**1) Comparability of cohorts on the basis of the design or analysis
 a) study controls for type of surgery or injury severity score for trauma patients
 b) study controls for comorbidities
 c) none of above  **Outcome**1) Assessment of outcome
 a) independent blind assessment
 b) record linkage
 c) self report
 d) no description
2) Was observation period long enough for outcomes to occur (*Details provided at the end)
 a) yes
 b) no
 c) no description
3) Adequacy of follow up of cohorts if applicable ( if not applicable^**^)
 a) complete follow up - all subjects accounted for
 b) subjects lost to follow up unlikely to introduce bias - ≥ 80% followed up or description
 provided of those lost
 c) follow up rate < 80% and no description of those lost
 d) no statement

Note: A study can be awarded a maximum of one star for each numbered item within the Selection and Outcome categories. A maximum of two stars can be given for Comparability.

| **Supplementary Table 1.** Methodology of *ex vivo* LPS stimulated cytokine production assays | | | | | | | |  |  |
| --- | --- | --- | --- | --- | --- | --- | --- | --- | --- |
| First author, year | Type of blood sample | Anti-coag | Dilution solution | Dilution ratio (blood: diluent) | Type of LPS | LPS conc. (ng/ml) | Incubation time (hours) | Method of measurement | Cytokines |
| Justus, 2017 | WB | NR | RPMI 1640 | NR | *E. coli* O55:B5 | 100 | 24 | FACS | TNFα |
| Flier, 2015 | WB | Lithium Heparin | NR | NR | *E. coli* O55:B5 | 10 | 2 | Bead-based immunoassay | TNFα, IL-6, IL-8 |
| Paraschos, 2015 | PBMC | Heparin* | RPMI 1640 | N/A | *E. coli* O55:B5 | 10 | 24 | ELISA | TNFα, IL-10, IL-17, IFNγ |
| Relja, 2015 | Monocytes | EDTA | RPMI 1640 | N/A | *E. coli* O127:B8 | NR | 24 | ELISA | IL-1β |
| Stoppelkamp, 2015 | WB | Heparin* | RPMI 1640 | 1:05 | *E. coli* O113:H10:K | 0.004-0.008^#^ | 8 | ELISA | IL-1β |
| Jones, 2014 | Monocytes | NR | NR | N/A | *E. coli* O26:B6 | 154 | 18 | Bead-based immunoassay | TNFα, IL-1β, IL-6, IL-8, IL-10, IL-12 |
| Kumpf, 2010 | Monocytes | Heparin* | NR | N/A | *E. coli* O155:B5 | 1 | NR | Bead-based immunoassay | TNFα, IL-6, IL-10 |
| Mokart, 2010 | Monocytes | NR | Isotonic saline | N/A | *E. coli* O55:B5 | NR | 16 | ELISA | IL-1Ra, IL-6, IL-10, IL-12p40 |
| Kirchhoff, 2009 | WB | Heparin* | RPMI 1640 | 1:10 | *E. coli* O55:B5 | 1000 | 4 | FACS | TNFα, IL-1β, IL-6, IL-8 |
| Wutzler, 2009 | WB | Heparin* | NR | NR | NR | NR | 24 | ELISA | IL-1β |
| Allen, 2006 | WB | Heparin* | RPMI 1640 | 1:01 | *Neisseria meningitides* | 10 | 24 | ELISA | TNFα, IL-6, IL-10 |
| Ploder, 2006 | WB | Lithium Heparin | NR | NR | NR | 0.5 | 4 | Chemi-luminescence | TNFα |
| Laudanski, 2004 | Monocytes | NR | RPMI 1640 | N/A | *E. coli* O111:B4 | NR | 16-18 | Bioassay | mTNFα |
| Spies, 2004 | WB | Ammonium heparin | NR | NR | NR | 0.5 | 4 | ELISA | IFNγ, IL-10 |
| Spolarics, 2003 | Monocytes | Heparin* | NR | N/A | *E. coli* O26:B6 | 5 | 4 | FACS | TNFα |
|  |  |  |  |  |  |  | 24 |  | IL-10 |
|  |  |  |  |  |  |  | 22 |  | IL-12^ |
| Heesen, 2002 | WB | Ammonium Heparin | RPMI 1640 | 1:01 | *Salmonella friedenau* | 100 | 4 | ELISA | IL-6 |
| Tashiro, 2001 | WB | NR | NR | NR | NR | 909 | 24 | ELISA | TNFα, IL-1α, IL-1β, IL-6, IFNγ, G-CSF |
| Majetschak, 2000 | WB | Ammonium Heparin | RPMI 1640 | 1:01 | *Salmonella friedenau* | 100 | 4 | ELISA | TNFα, IL-6, IL-8 |
| Majetschak, 2000 | WB | Ammonium heparin | RPMI 1640 | 1:01 | *Salmonella friedenau* | 100 | 4 | ELISA | TNFα |
| Riese, 2000 | WB | Heparin* | NR | NR | *E. coli* O55:B5 | 10 | 5 | ELISA | TNFα, IL-6 |
| Van Bokhorst, 2000 | WB | Heparin* | NR | NR | *E. coli** | 100 | 4 | ELISA | TNFα, IL-6 |
| Flach, 1999 | WB | Ammonium heparin | RPMI 1640 | 1:01 | *Salmonella friedenau* | 100 | 4 | ELISA | TNFα, IL-6, IL-8 |
| Ziegenfuss, 1999 | WB | NR | RPMI 1640 | 1:05 | *E. coli* O111:B4 | 1000 | 24 | Bioassay | TNFα, IL-6 |
|  |  |  |  |  |  |  |  | ELISA | IL-1, IL-10 |
| Schluter, 1991 | PBMC | Heparin* | RPMI 1640 | N/A | *E. coli* O127:B8 | NR | 20 | ELISA | IL-6 |
| Wood, 1984 | Monocytes | Heparin* | MEM | N/A | *E. coli* O55:B5 | 1000 | 24 | Bioassay | IL-1 |

*Did not further specify for type. #Value estimated from 0.04 EU/ml. ^IL-12 pre-incubated with 10ng/ml IFNγ for 2 hours then incubated with LPS. Anti-coag = anti-coagulant. Conc = concentration. WB = Whole blood. PBMC = peripheral blood mononuclear cell. EDTA = Ethylenediaminetetraacetic acid. RPMI = Roswell Park Memorial Institute medium. MEM = Minimum essential medium. *E. coli = Escherichia coli*. FACS = Fluorescence-activated cell sorting. ELISA = enzyme linked immunosorbent assay. sTNFα = secreted TNFα. mTNFα = membrane-associated TNFα. NR = not reported. N/A = not applicable.

## **Supplementary Table 2.** Overall quality scores for articles as determined by the modified Newcastle-Ottawa scale

| First author, year | Selection | Comparability | Outcome |
| --- | --- | --- | --- |
| Justus, 2017 |  |  |  |
| Flier, 2015 |  |  |  |
| Paraschos, 2015 |  |  |  |
| Relja, 2015 |  |  |  |
| Stoppelkamp, 2015 |  |  |  |
| Jones, 2014 |  |  |  |
| Kumpf, 2010 |  |  |  |
| Mokart, 2010 |  |  |  |
| Kirchhoff, 2009 |  |  |  |
| Wutzler, 2009 |  |  |  |
| Allen, 2006 |  |  |  |
| Ploder, 2006 |  |  |  |
| Laudanski, 2004 |  |  |  |
| Spies, 2004 |  |  |  |
| Spolarics, 2003 |  |  |  |
| Heesen, 2002 |  |  |  |
| Tashiro, 2001 |  |  |  |
| Majetschak, 2000 |  |  |  |
| Majetschak, 2000 |  |  |  |
| Riese, 2000 |  |  |  |
| Van Bokhorst, 2000 |  |  |  |
| Flach, 1999 |  |  |  |
| Ziegenfuss, 1999 |  |  |  |
| Schluter, 1991 |  |  |  |
| Wood, 1984 |  |  |  |

## **Supplementary Figure 1:** Funnel plot of the articles that investigated the association between LPS stimulated IL-6 and risk of inflammatory complications

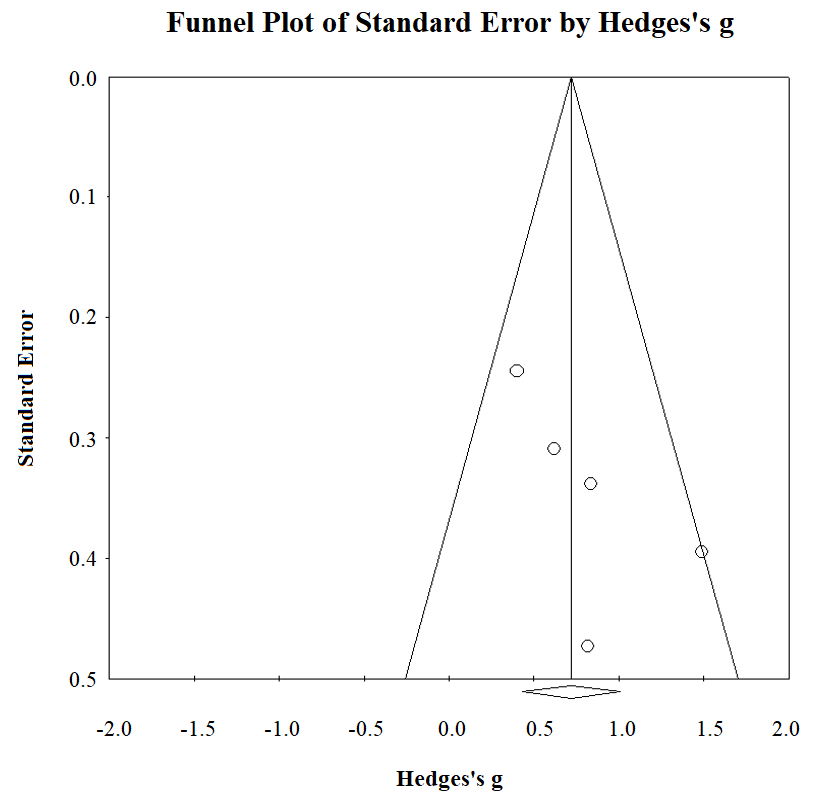


**Figure 1:** Funnel plot of the SMD meta-analysis of published (n=5) articles. Each plotted point represents the standardised mean difference between IL-6 stimulated LPS measurements. The white triangle represents the region where 95% of the data points would lie in the absence of publication bias. The vertical line represents the average SMD in the meta-analysis.
