# Supplementary figures and images for: Immune capacity determines outcome following surgery or trauma: a systematic review and meta-analysis
Source: Eur J Trauma Emerg Surg. 2019 Nov 28;46(5):979–91. doi: 10.1007/s00068-019-01271-6 (PMC7593308; doi:10.1007/s00068-019-01271-6)

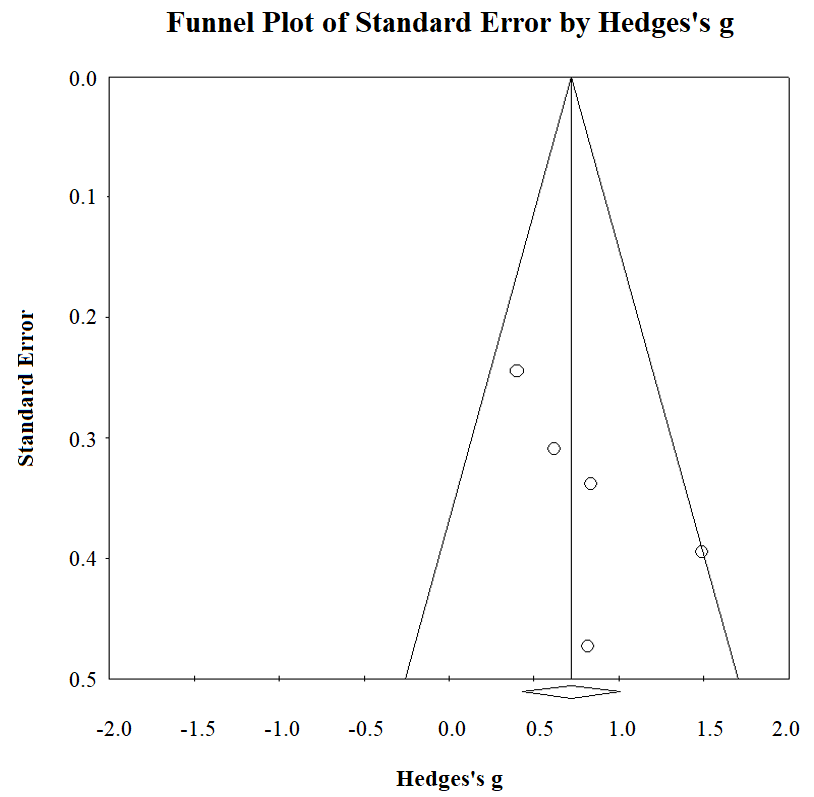

Supplement: Supplementary file 2 — Supplementary file2 (TIFF 44 kb) [file 68_2019_1271_MOESM2_ESM.tiff]
